# The improvement of *Coreopsis tinctoria* essential oil on learning and memory impairment of d-galactose-induced mice through Nrf2/NF-κB pathway

**DOI:** 10.3389/fphar.2022.994705

**Published:** 2022-08-24

**Authors:** Yan Qu, Yingxue Guo, Wenpeng Li, Hongkuan Shen, Jiwen Cui, Jinlian Li, Jiguang Liu, Dongmei Wu

**Affiliations:** ^1^ Key Laboratory of Microecology-Immune Regulatory Network and Related Diseases School of Basic Medicine, Jiamusi University, Jiamusi, China; ^2^ College of Jiamusi, Heilongjiang University of Chinese Medicine, Jiamusi, China; ^3^ College of Pharmacy, Jiamusi University, Jiamusi, China; ^4^ School of Stomatology, Jiamusi University, Jiamusi, China; ^5^ Jiamusi Inspection and Testing Center, Jiamusi, China

**Keywords:** coreopsis tinctoria, essential oil, D-galactose, learning and memory impairment, Nrf2/NF-κB pathway

## Abstract

Essential oil of *Coreopsis tinctoria* (EOC) is a essential substance extracted from *Coreopsis tinctoria* with the excellent anti-oxidant effect. However, it is still unclear whether EOC can improve learning and memory impairment and its mechanism. The purpose of this study was to investigate the effect of EOC on learning and memory impairment induced by D-galactose (D-gal) in mice and reveal its mechanism. The composition of EOC was analyzed by GC-MS, and the results showed that the highest content was D-limonene. The follow-up experiments were conducted by comparing EOC with D-limonene. The aging model was established by subcutaneous injection of D-gal, and donepezil, D-limonene and EOC were given by intragastric administration. It was found that EOC and D-limonene significantly improved learning and memory impairment induced by D-gal through the Morris water maze and step-through tests. Pathological and biochemical analysis showed that the hippocampal morphologic of mice was damage and the activities of superoxide dismutase (SOD) and glutathione peroxidase (GSH-Px) induced by D-gal were decreased, while the content of malondialdehyde (MDA) was increased, while EOC and D-limonene could reverse the morphological changes and reduce oxidative damage. In addition, EOC and D-limonene significantly increased body weight and organ coefficients, including liver, spleen and kidney. Moreover, EOC and D-limonene improved the expression of nuclear factor E2 related factor 2 (Nrf2) pathway and inhibited nuclear transcription factors-κB (NF-κB) pathway. In summary, the results showed that EOC and D-limonene could improve learning and memory impairment induced by D-gal through Nrf2/ NF-κB pathway. It was clear that as a mixture, EOC was better than D-limonene on improving learning and memory impairment.

## 1 Introduction

As the average life span of the global population is prolonged in the 21st century, aging has become one of the most important social problems (Li et al., 2019; O’Meara, 2020). Learning and memory impairment is one of the important manifestations of brain aging, and aging has become an important factor of these cognitive impairments inevitably (Beal, 1995; Molloy et al., 2014). Learning and memory impairment is the main feature of the aging population, which brings a heavy burden to the family and society. Therefore, it is urgent to find drugs that can effectively improve learning and memory impairment. So far, the mechanism of aging is not fully understood, while the excessive production of reactive oxygen species is considered to be an important factor on improving aging and plays an important role in the study of brain aging (Annunziato et al., 2002; Lee et al., 2004). Oxidative stress is a stress response caused by excessive ROS in the body, which is the result of imbalance between oxidation and antioxidation, and eventually leads to oxidative damage of cell membrane and DNA (Risom et al., 2005; Kryston et al., 2011). It can be inferred that oxidative stress is the key factor of learning and memory impairment in the elderly (Feng et al., 2012). Therefore, inhibiting oxidative stress may be a potential method to improve learning and memory impairment.

Nuclear factor E2 related factor 2 (Nrf2) is the basis of defense against ROS and one of the key antioxidant pathway proteins (Nguyen et al., 2009; Linker et al., 2011). Nrf2 can induce the expression of enzymes that regulate activated antioxidant response elements such as superoxide dismutase (SOD) and glutathione peroxidase (GSH-Px) and heme Oxygenase-1 (HO-1) (Long et al., 2017; Ma et al., 2020). The Nrf2 expression decreases and the ROS content increases in the patients’ hippocampus with neurodegenerative diseases which increases the sensitivity of neurons to oxidative stress and eventually leads to brain damage (Ramsey et al., 2007; Scuderi et al., 2020). Nuclear transcription factors-κB (NF-κB) is the main transcription factor activated by oxidative stress. It is caused by the increased activation of ROS, which causes a large amount of secretion of downstream inflammatory factors (Zhang et al., 2012; Moniruzzaman et al., 2018). Therefore, drugs targeting oxidative stress-related proteins NF-κB and Nrf2 are considered to have the potential to improve learning and memory impairment.

*Coreopsis tinctoria Nutt.* (*C. tinctoria Nutt., Coreopsis tinctoria*) is a chrysanthemum plant, which is native to North America and has been now widely planted all over the world. It is usually used as tea in the folk, and it has anti-oxidant, anti-aging, anti-hyperlipidemia and anti-cancer effects (Dias et al., 2010; Guo et al., 2015; Ren et al., 2018). Especially, the essential oil of *Coreopsis tinctoria* (EOC) is famous for the excellent antioxidant effect (Yao et al., 2016). D-limonene is the most important component in EOC, and its content is as high as 50.961%. A large number of studies have shown that D-limonene can exert its antioxidant effect by restoring antioxidant enzymes and reducing acetylcholinesterase activity, reducing lipid peroxidation level (Szwajgier and Baranowska-Wojcik, 2019; Boiangiu et al., 2020; Sanchez-Martinez et al., 2020). It is speculated that EOC, which is mainly composed of D-limonene, may have a good performance on improving learning and memory impairment. Therefore, a comparative study was conducted on the improvement effect of EOC and D-limonene on learning and memory impairment through Nrf2/NF-κB signal pathway in this paper. It is clear that as a mixture, EOC can improve learning and memory impairment better than D-limonene, which provides a theoretical basis for the development of EOC for the prevention or treatment on learning and memory impairment, and expands its application in natural medicine, pharmacy and food industry.

## 2 Materials and methods

### 2.1 Reagents

D-limenone (puity≥98%) and D-gal (puity≥99%): Sigma-Aldrich (St. Louis, United States). Donepezil (puity≥98%): Shanghai Yuanye Biotechnology Co., LTD. (Shanghai, China). All analysis kits: Nanjing Jiancheng Bioengineering Institute (Nanjing, China). The following antibodies: Beijing Bioss Biotechnology Co., LTD. (Beijing, China) at a dilution ratio of 1: 1,000: anti-Iκκβ (bs-4880R), anti-NF-κB (bs-3485R), anti-TNF-α (bs-0078R), anti-IL-1β (bs-0812R), anti-Keap1 (bs-4900R), anti-Nrf2 (bs-1074R), and anti-HO-1 (bs-23667R). Anti-GAPDH (bs-0061R) at a dilution ratio of 1:10000 and IgG/HRP antibody (bs-0293P) at a dilution ratio of 1:2000.

### 2.2 Animals and administration

**FIGURE 1:**
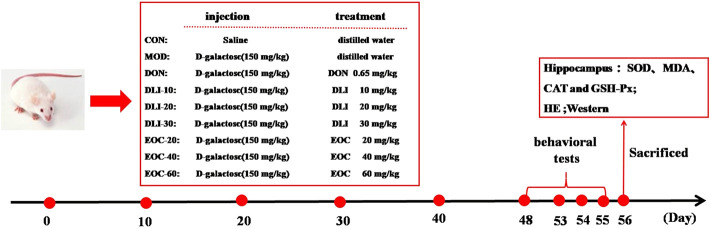
Animal experiment program.

Ninety male Kunming mice (body weight, 18-22 g; age, 6-8 weeks) were purchased from Changchun Yisi Experimental Animal Technology Co.,Ltd. (Changchun, China). The mice were placed under standard conditions with a set temperature of 22 ± 2°C, humidity of 55 ± 10%, and 12 h/12 h day-night ratio. After 7 days of normal feeding, mice were randomly divided into nine groups, with 10 mice in each group: control group (CON), D-gal group (MOD), donepezil group (DON), DLI dose groups (DLI-L/-M/-H), EOC dose groups (EOC-L/-M/-H). Except for CON group, the mice in other groups were injected with 150 mg/kg D-gal subcutaneously, and CON group was injected with the same amount of normal saline. At the same time, the mice in each group were given intragastric administration once a day, in which Donepezil 0.65 mg/kg, DLI-L/-M/-H dose groups were 10, 20 and 30 mg/kg, and EOC-L/-M/-H dose groups were 20, 40 and 60 mg/kg respectively. CON group and MOD group received the same amount of distilled water, and all mice were treated with different drugs for 56 days. Behavioral tests were performed from the 48^th^ to the 55^th^ day after administration. On the 56^th^ day, mice tissues were collected for testing (Fig. 1).

### 2.3 Extraction of EOC and principal component analysis

*Coreopsis tinctoria* that was used for this experiment was planted in Jiamusi University Science Park. EOC was extracted dried flowers from *Coreopsis tinctoria* by steam distillation for 3 h with double-distilled water as the solvent. EOC was dried by anhydrous sodium sulfate and filtered to obtain the sample; Chromatographic column: specification of DB-5MSTTD elastic quartz capillary column: 30 m × 0.25 mm, 0.25 μm; Injection port temperature: 240°C; Injection mode: split injection, split ratio 50:1, injection volume 0.1 μL; Column flow rate: 1.0 mL/min; Programmed temperature rise of column temperature: the initial temperature is 130°C, keep it for 5 mins, and program temperature rise to 230°C at the rate of 5°C/min for 10 min.

### 2.4 Morris water maze test

The Morris water maze is a behavioral experimental method which is used to test animals for learning and memory abilities. The tank used in the experiment was divided into four areas. The hidden platform was in the center of the third area. The water level was about 1 cm higher than the hidden platform. The water temperature was controlled at 22-24°C. Each time the mouse was placed at an equal distance from the four quadrants, set the time to find the hidden platform to 60 s, and stayed for 15 s after finding the hidden platform. The mice which couldn’t find the platform were recorded for 60 s and led to the platform in order to stay for 15 s. The time from mouse released to finding the hidden platform became the escape latency. After 5 days of training, the space exploration experiment would be carried out. Then the hidden platform was taken out of the water tank, and then each mouse swam at will for 60 s. The camera hanging directly above the water tank recorded the action path of the mice.

### 2.5 Step-through test

The mice were trained for the step-through test to evaluate memory. The setup for the step-through test was comprised of a light room and a dark room. Entrying into the dark room was set off a 36 V electric shock. The mice were placed in the light room at first. Because mice preferred the dark, they ran into the dark room, where they received an electric shock that resulted in them escaping back into the light room. The experiment was conducted for 5 min for each mouse. After a day of training, the formal experiment was conducted. The escape latency was the time required to first enter the dark room to receive a shock. The escape latency and the number of times entered the dark room in the formal experiment were recorded and statistically processed.

### 2.6 Body weight measurement and organ coefficients

All mice were weighed every Monday during the experiment. Water and food were prohibited 12 h before sacrificed. The organ coefficients were calculated as the weight of the organ (liver, spleen, or kidney) (mg) divided by body weight of the mouse (g).

### 2.7 Detection of MDA, SOD, and GSH-Px in the mice hippocampus

After the mice were sacrificed, the hippocampus was isolated and homogenized in ice cold sterile PBS. The homogenate was centrifuged in a told cryogenic centrifuge at 12000 r/min for 8 min at 4°C. After centrifugation, the supernatant was taken according to the instructions of the kits.

### 2.8 Histopathological examination of the hippocampus

The tissues were isolated from mice, fixed with 4% paraformaldehyde, and dehydrated with ethanol. The tissues were embedded in paraffin wax and cut with a slicer at a thickness of 4 μm. The sections were then dewaxed, rehydrated, treated with an antiseptic, and stained with hematoxylin-eosin staining (HE). Histopathological changes were evaluated with a light microscope. The hippocampus was observed at magnifications of ×400.

### 2.9 Western blot analysis

After hippocampal tissue was obtained, it was fully lysed in RIPA buffer and centrifuged at 12000 r/min at 4°C for 8 min. The supernatant was added to the loading buffer and boiled at 100°C for 8-10 min. Samples (20 g/protein) were transferred to polyvinylidene fluoride membranes after separation by 10% SDS-PAGE. After addition of primary antibody, they were incubated at 4°C overnight. The secondary antibody was incubated the next day for 1 h after the primary antibody incubation at room temperature. Reference GAPDH was the reference. The membranes were visualized using the ECL reagent and analyzed using a gel imaging system (Tianneng technology instrument, Shanghai, China).

### 2.10 Statistical analysis

Statistical analysis of the experimental data was performed using the IBM SPSS 16.0. The experimental data were presented as mean ± S.D. and analyzed by one-way ANOVA. The *t*-test was used for comparison of groups. *p* values <0.05 or *p* values <0.01 was considered to indicate statistical significance.

## 3 Results

### 3.1 Analysis of EOC composition

A total of 36 chromatographic peaks were detected by GC-MS, and 23 compounds, including 19 alkenes, one aldehyde, one phenol, one ether, and one naphthalene, were identified. The main components were D-limonene, α-phellandrene, (1R)-α-pinene, and β-myrcene (Table 1), which accounted for 50.961%, 5.717%, 5.226%, and 5.215% of the total content. D-Limonene was the most important component of EOC, and other components were trace components, which played a major role in the overall efficacy. The follow-up experiments were conducted by comparing it with D-limonene.

**TABLE 1:** Analysis of main components of EOC.

No.	Retention time (min)	Chemical composition	Relative content (%)	Molecular formula
1	7.465	(1R)-α-pinene	5.226	C_10_H_16_
2	8.612	β-Phellandrene	1.782	C_10_H_16_
3	9.173	β-Myrcene	5.215	C_10_H_16_
4	9.674	α-Phellandrene	5.717	C_10_H_16_
5	10.579	limonene	50.961	C_10_H_16_
6	10.905	(Z)-13,7-dimethyl-3,6-octatriene	0.352	C_10_H_16_
7	11.177	g-Terpinene	0.261	C_10_H_16_
8	11.521	(E)-3,7-dimethyl-2,6-Octadienal	0.266	C_10_H_16_O
9	12.046	Phenol,2,3,5,6-tetramethyl-	0.419	C_10_H_14_O
10	13.018	3,4-Dimethyl-2,4,6-octatriene	2.058	C_10_H_16_
11	13.241	Limonene oxide, trans	0.410	C_10_H_16_O
12	15.565	2-methoxy-4-methyl-1-(1-methylethyl)-Benzene	0.344	C_11_H_16_O
13	17.852	2,2,8-Trimethyltricyclo [6.2.2.01,6]dodec-5-ene	0.258	C_15_H_24_
14	18.317	thujopsene-β	2.724	C_15_H_24_
15	18.999	Copaene	0.312	C_15_H_24_
16	19.976	Caryophyllene	1.226	C_15_H_24_
17	20.284	2,6-dimethyl-6-(4-methyl-3-pentenyl)bicyclo [3.1.1]hept-2-ene	4.088	C_15_H_24_
18	20.743	α-Caryophyllene	0.345	C_15_H_24_
19	21.202	(+)-b-Himachalene	0.594	C_15_H_24_
20	21.334	Germacrene D	2.938	C_15_H_24_
21	22.004	d-Cadinene	0.255	C_15_H_24_
22	23.308	Caryophyllene-oxide	1.288	C_15_H_24_O
23	25.288	Naphthalene,1-(2-propen-1-yl)	0.582	C_13_H_12_

### 3.2 Effects of EOC and DLI on the morris water maze test in mice

**FIGURE 2:**
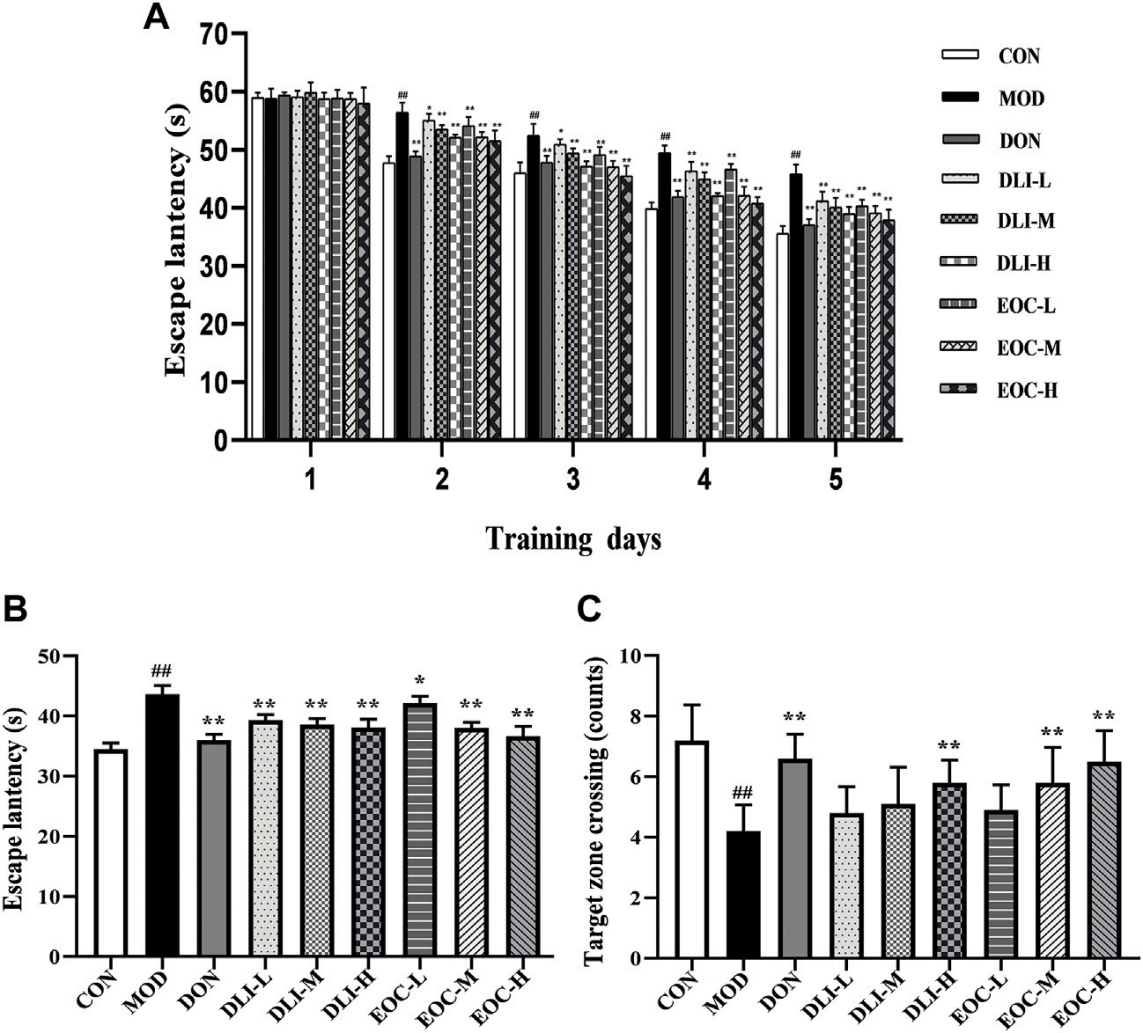
Effect of EOC and DLI on the Morris water maze test in mice (*n* = 10). **(A)** The escape latency in the positioning navigation experiment. **(B)** The escape latency of first entry into the target quadrant in the space exploration experiment. **(C)** The number of entries into the target quadrant in the space exploration experiment. ^#^ *p* < 0.05; ^##^ *p* < 0.01 vs. the CON group; *p <0.05, **p <0.01 vs. the MOD group.

Morris water maze test is a classic method to study learning and memory ability, divided into the positioning navigation and space exploration experiments. The results of the positioning navigation experiment are shown that the escape latency of mice in each experimental group was gradually shortened with the increase of training days (Figure 2A) The escape latency in MOD group was significantly longer compared with CON group (*p* <0.05); the escape latency in DLI and EOC groups were shortened compared with MOD group in a dose-dependent manner (*p* <0.05 or *p* < 0.01). The D-limonene content of EOC-M group was similar to that of DLI-M group, but the escape latency in EOC-M group was significantly shortened than that in DLI-M group, and close to that in DLI-H group. The results of the space exploration test showed that the target quadrant time and the number of crossing the platform in MOD group were significantly shortened than those in CON group after the platform was removed (Figures 2B,C) (*p* <0.01). Compared with MOD group, DLI groups showed no significant difference in the target quadrant time (*p* <0.01), but the number of crossing the platform was increased in a dose-dependent manner. The target quadrant time and the number of crossing the platform in EOC groups were increased in a dose-dependent manner. There was no significant difference between EOC-M group and DLI-H group, suggesting that other little components in essential oil may play a synergistic role in ameliorating learning and memory impairment.

### 3.3 Effects of EOC and DLI on the step-through test in mice

**FIGURE 3:**
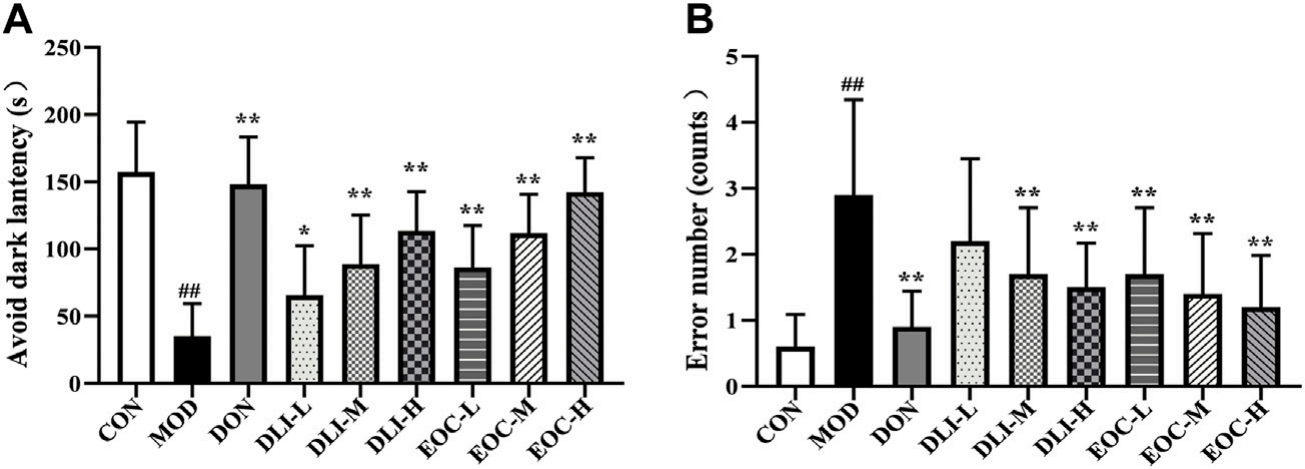
Effect of EOC and DLI on the step-through maze test in mice (*n* = 10). **(A)** Latency to first entry into the dark room. **(B)** The number of entries into the dark room. ^#^ *p* < 0.05; ^##^ *p* < 0.01 vs. the CON group; *p < 0.05, **p < 0.01 vs. the MOD group.

Step-through test is to study memory ability. The results of the step-through test showed that compared with CON group, the escape latency of mice in MOD group was significantly shortened and the number of errors was increased (Figure 3) (*p* <0.01). Compared with MOD group, the escape latency in DLI and EOC groups were shortened in a dose-dependent manner (*p* <0.05 or *p* < 0.01); the number of errors in DLI and EOC groups were shortened in a dose-dependent manner. In the escape latency and number of errors, the effect of EOC-M group was similar to that of DLI-H group, and there was no significant difference, indicating that other little components in essential oil had synergistic effect on ameliorating memory ability.

### 3.4 Effects of EOC and DLI on body weight gain and organ coefficients in mice

**FIGURE 4:**
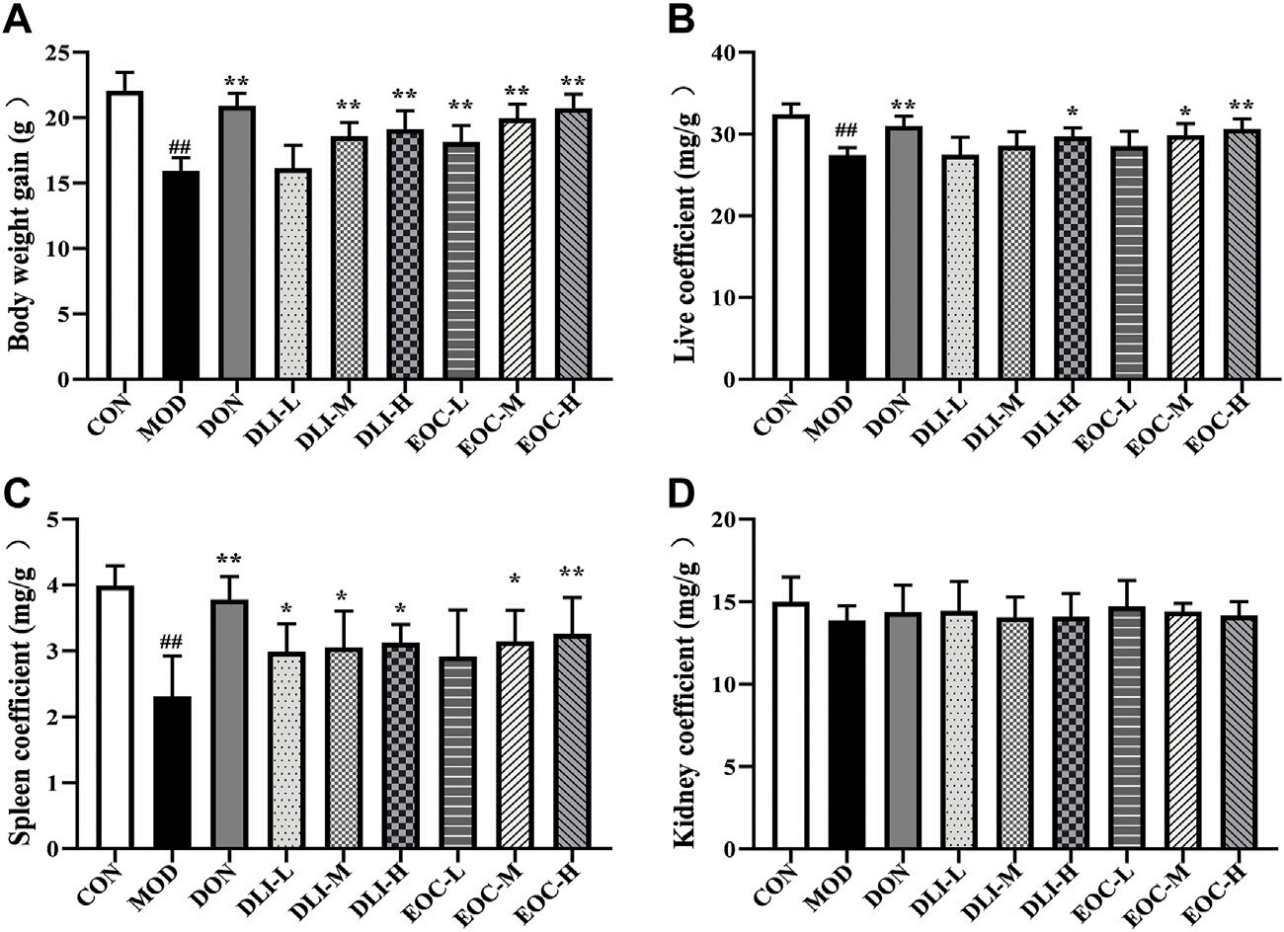
Effect of EOC and DLI on body weight gain and organ coefficients (*n* = 5). **(A)** Body weight gain; **(B,C,D)** Liver coefficient, spleen coefficient, and kidney coefficient. ^#^ *p* < 0.05; ^##^ *p* < 0.01 vs. the CON group; *p <0.05, **p <0.01 vs. the MOD group.

Aging can lead to slow weight gain and degeneration of immune related organs (such as spleen), and the coefficients of these organs are usually used as markers to assess the effectiveness of aging mouse. Compared with CON group, the weight gain of mice in MOD group was decreased significantly (Figure 4A) (*p* <0.01); Compared with MOD group, the weight gain of mice in DLI and EOC groups was increased in a dose-dependent manner. There was no significant difference in body weight gain between EOC-M group and DLI-H group, and the effect was similar. Compared with CON group, the liver coefficient and spleen coefficient in MOD group were significantly decreased (Figures 4B,C); The liver and spleen coefficient in DLI and EOC groups were higher than those in MOD group in a dose-dependent manner. There was no significant difference in liver coefficient and spleen coefficient between EOC-M group and DLI-H group, and the effect was similar, indicating that other little components in essential oil played a synergistic role in ameliorating organ aging. There was no significant difference in kidney coefficient among groups, indicating that DLI and EOC had no significant effect on mouse kidney (Figure 4D).

### 3.5 Effects of EOC and DLI on brain SOD, MDA and GSH-Px in mice

**FIGURE 5:**
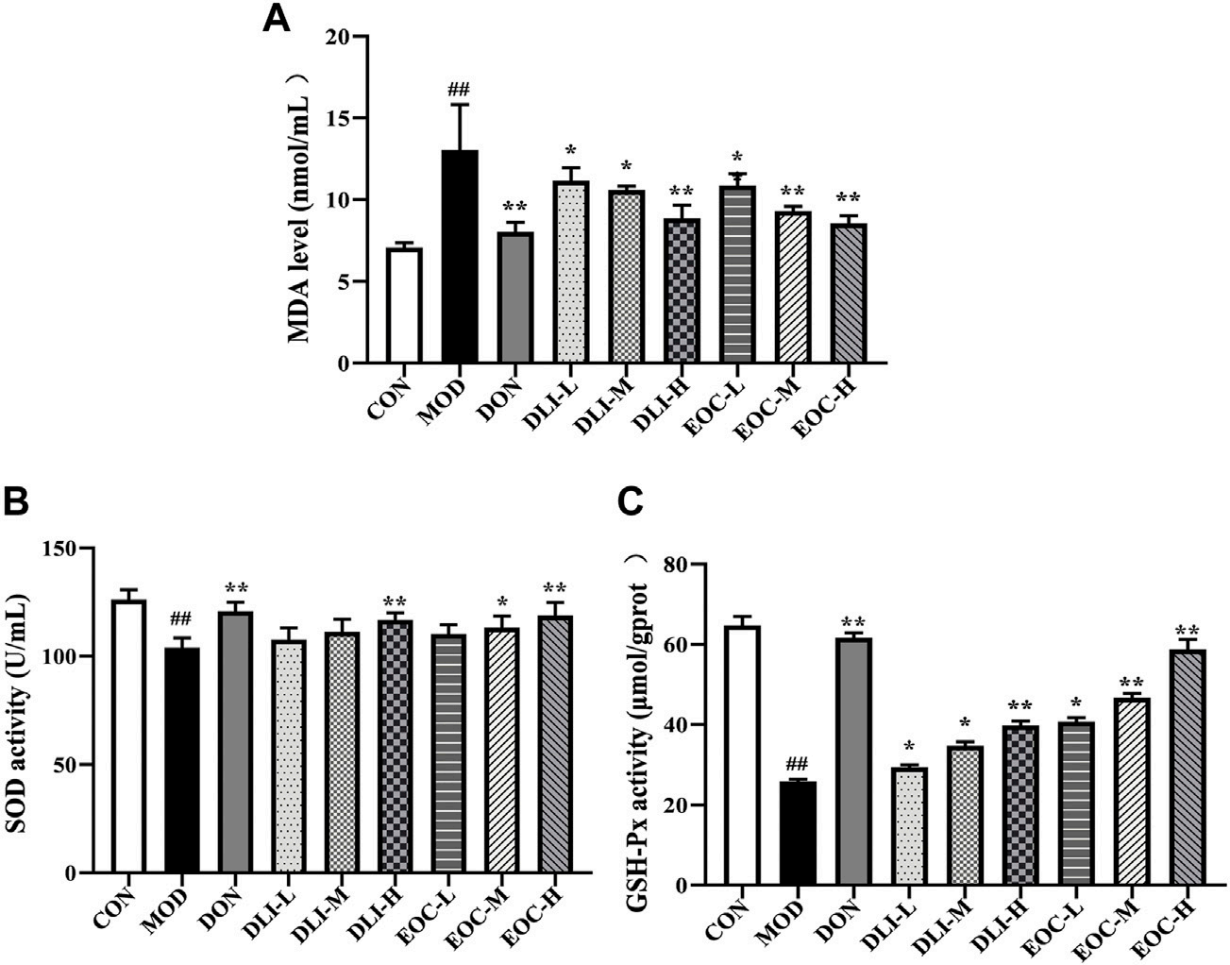
Effects of EOC and DLI on brain SOD, MDA and GSH-Px in mice (*n* = 5). **(A)** MDA. **(B)** SOD. **(C)** GSH-Px. ^#^ *p* < 0.05; ^##^ *p* < 0.01 vs. the CON group; *p <0.05, **p <0.01 vs. the MOD group.

The contents of SOD, MDA and GSH-Px in hippocampus were determined to reflect the level of oxidative stress. Compared with CON group, MDA level in MOD group was significantly increased (*p* <0.01), while SOD and GSH-Px activities were significantly decreased (Figure 5) (*p* <0.01). Compared with MOD group, MDA levels in DLI and EOC groups were decreased in a dose-dependent manner (*p* <0.05 or *p* <0.01), while SOD and GSH-Px were increased in a dose-dependent manner (*p* <0.05 or *p* <0.01). There was no significant difference in the contents of SOD, MDA and GSH-Px between EOC-M group and DLI-H group, which indicated that other components of EOC had synergistic effect on antioxidation.

### 3.6 Effects of EOC and DLI on the hippocampus in mice

**FIGURE 6:**
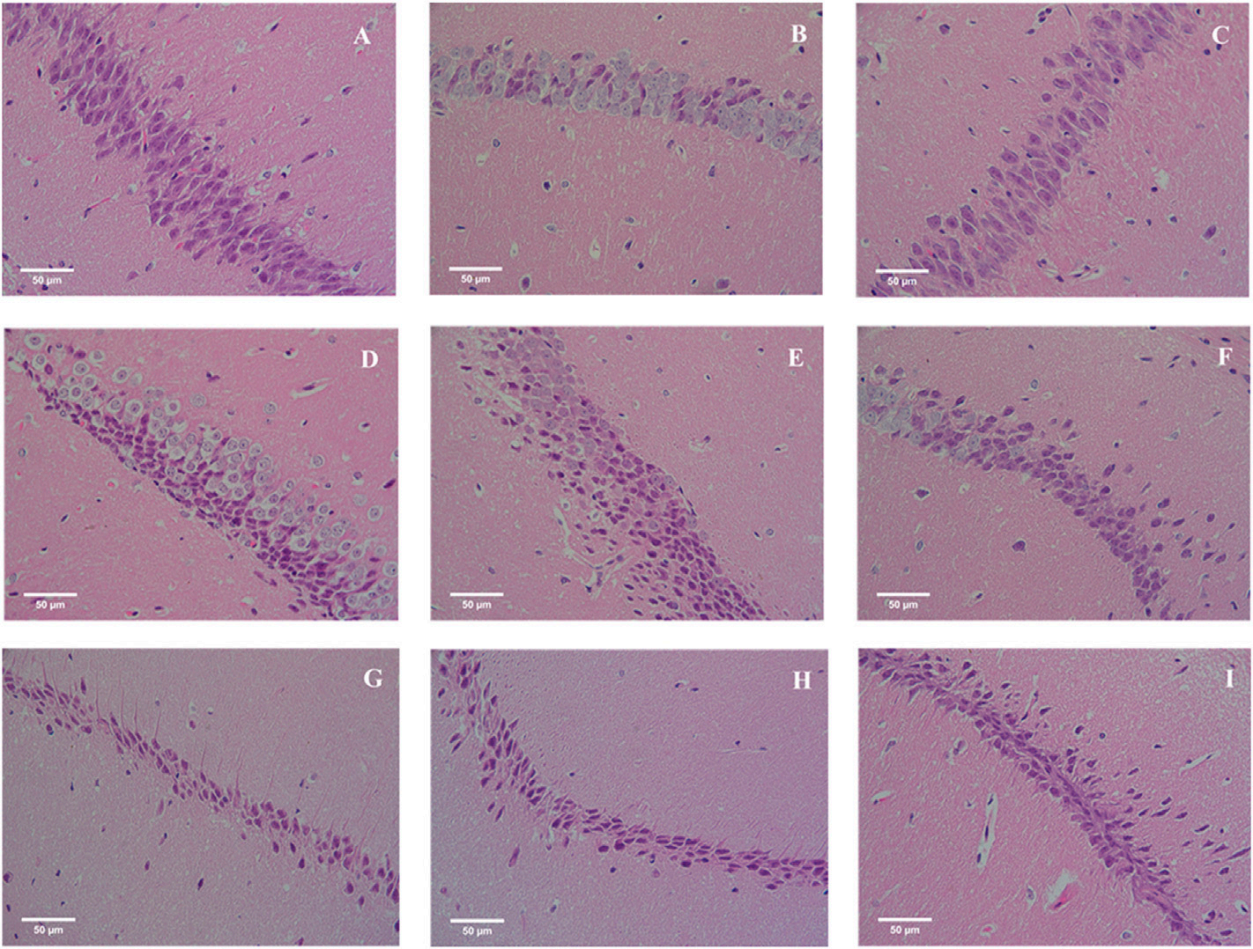
Effect of EOC on the hippocampus in mice (HE, original magnification, ×400). **(A)** CON. **(B)** MOD. **(C)** DON. **(D)** DLI-L. **(E)** DLI-M. **(F)** DLI-H. **(G)** EOC-L. **(H)** EOC-M. **(I)** EOC-H.

Hippocampus is responsible for memory storage, conversion and orientation. The pathological results of mice hippocampus were shown in Figure 6. The neurons of hippocampus in CON group were arranged tightly and orderly, and the nuclei were large and round. The number of neurons in MOD group was less than that in CON group, and the arrangement was sparse and irregular. Compared with MOD group, neurons in DLI and EOC groups were increased, arranged closely and relatively regularly, especially EOC-H, indicating that EOC and DLI groups could reduce the nerve injury induced by D-gal, and the effect of EOC in reducing nerve injury was better than DLI.

### 3.7 Effect of EOC and DLI in the hippocampus of mice through the nrf2/NF-κb pathway

**FIGURE 7:**
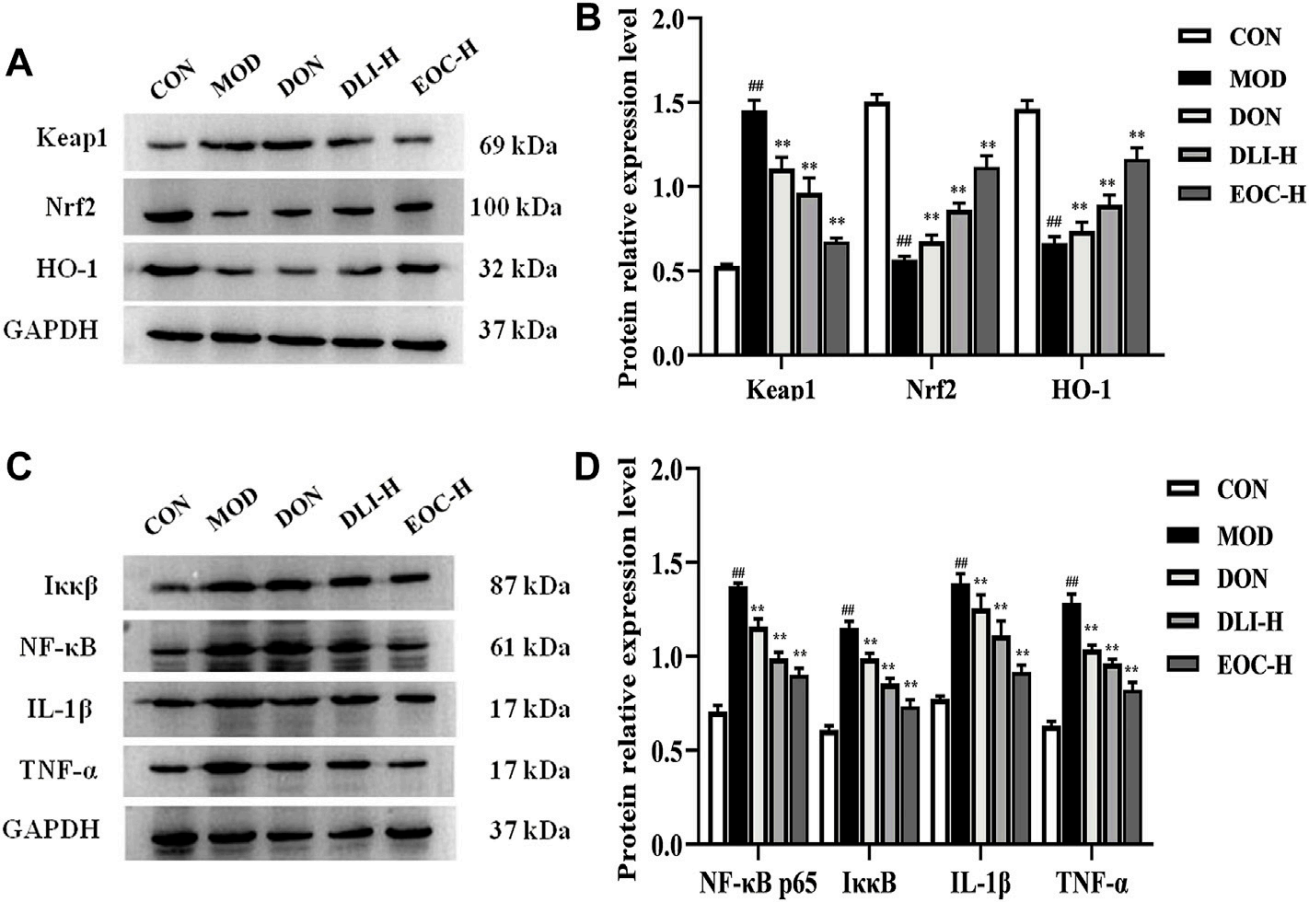
Effect of EOC in the hippocampus via Nrf2/ NF-κB (*n* = 5). **(A,C)** Western blot images. **(B,D)** Relative expression of the protein. ^#^ *p* < 0.05; ^##^ *p* < 0.01 vs. the CON group; *p < 0.05, **p < 0.01 vs. the MOD group.

The expression levels of related protein factors Nrf2 and NF-κB in the hippocampus were shown in Figure 7. As shown in Figures 7A,B, compared with CON group, the expression of Nrf2 and HO-1 in MOD group decreased significantly, and the expression of Keap1 increased significantly (*p*<0.01). Compared with MOD group, the expression of Nrf2 and HO-1 in DLI-H group and EOC-H group increased, and the expression of Keap1 decreased (*p* <0.01). Moreover, the expression levels of Nrf2 and HO-1 in EOC-H group were higher than those in DLI-H group, indicating that other little components in essential oil had synergistic effect on Nrf2 pathway. As shown in Figures 7C,D, compared with CON group, the expressions of IκκB, NF-κB, IL-1β and TNF-α in MOD group increased significantly (*p* <0.01). Compared with MOD group, the expressions of IκκB、NF-κB、IL-1β and TNF-α in DLI-H group and EOC-H group decreased (*p* <0.01). Furthermore, the expression levels of IκκB, NF-κB, IL-1β and TNF-α in EOC-H group were lower than those in DLI-H group, indicating that other little components in essential oil had synergistic effect on NF-κB pathway. In general, DLI and EOC played a neuroprotective role through the Nrf2/NF-κB signaling pathway, and EOC was superior to DLI.

## 4 Discussion

Brain aging is a natural and irresistible process, which is manifested by learning and memory impairment (Calhoun et al., 2008; Fukui et al., 2010). Oxidative stress caused by excessive production of ROS plays an important role in the pathogenesis of age-related neurodegenerative diseases (Yeung et al., 2020). A large number of experimental evidences show that antioxidants are effective methods to prevent brain aging by reducing oxidative stress (Mariani et al., 2005; Li et al., 2010). Among these antioxidants, essential oil is particularly prominent (Li et al., 2007).

*Coreopsis tinctoria* is a chrysanthemum plant, and it is commonly used as tea in folk. It has functions of anti-oxidation, anti-hypertensive, anti-cancer and so on. In this study, the main components of EOC detected by GC-MS were D-limonene (50.961%), α-phellandrene (5.717%), (1R)-α-pinene (5.226%) and β-myrcene (5.215%), all of which have good antioxidant effects (Soottitantawat et al., 2004; Kartal et al., 2007; Bajpai et al., 2009; Lachenmeier, 2010). D-Limonene not only has the highest content in EOC, but also has good antioxidant effect and protective effect on neurodegenerative diseases (Eddin et al., 2021). These results show that EOC contains a large number of olefins, especially D-limonene, so EOC may be a potential drug to improve learning and memory impairment. In the follow-up experiments, EOC and D-limonene were compared to explore the effect of EOC on learning and memory impairment.

Excessive D-gal is transformed into excessive ROS, which further accelerates the aging process (Zhang et al., 2015; Zhang et al., 2020). Aging diseases are often associated with weight gain, liver, spleen and kidney coefficient are also considered to be important indicators of aging (Lee et al., 2010). With aging, the function of organs will gradually decline. As the detoxification organ of the human body, the liver will also change with age. Aging can also cause changes in immune organs, such as spleen. In our study, mice were subcutaneously injected with D-gal (150 mg/kg) for 8 weeks, and the weight gain, liver and spleen coefficient in MOD group decreased significantly. The mice showed learning and memory impairment, slow movement and lethargy, which suggested that the aging model was successfully induced. However, EOC and DLI groups significantly increased the weight gain, liver and spleen coefficient, and improved their mental state and mobility. Because EOC and DLI could basically restore normal weight gain and delay liver and spleen atrophy, it could be considered that EOC and DLI could help delay aging, and the improvement effect of EOC was better than that of DLI. Moreover, EOC and DLI had no effect on kidney coefficient, indicating that EOC and DLI had no obvious kidney toxicity.

With the aggravation of aging, the rapid increase of ROS in the body leads to oxidative damage of cell membrane and DNA (Ruiz-Ramos et al., 2009; Rodríguez-Vargas et al., 2012). The brain lacking antioxidant defense mechanism is very vulnerable to ROS attack, which intensifies the oxidative damage of the brain, and then shows obvious learning and memory impairment (Liu et al., 2012; Baghcheghi et al., 2018). Both the Morris water maze and step-through tests are classical behavioral methods to investigate learning and memory, which can directly observe the behavior of mice (D`Hooge and De Deyn, 2001; Micale et al., 2010). Morris water maze results showed that the escape latency of platform searching in EOC and DLI groups were shortened in the positioning navigation experiment, increased the target quadrant time and the number of crossing the platform. EOC and DLI decreased the number of errors and time in dark room in a dose-dependent manner in the step-through test. According to the previous reports of behavioral tests, long-term use of D-gal could lead to learning and memory impairment (Li et al., 2021; Zhong et al., 2020). Through the Morris water maze and step-through tests, it was observed that EOC and DLI could improve learning and memory impairment, and the improvement effect of EOC was better than that of DLI. The results suggested that other components in EOC had synergistic effects on improving learning and memory impairment.

Hippocampus is an important structure that integrates short-term memory into long-term memory and spatial memory for navigation (Falkenberg et al., 1992; Howard et al., 1999). Histopathological results also confirmed the significant loss and irregular arrangement of hippocampal neurons induced by D-gal. After EOC and DLI treatment, the pathological damage was reduced. The improvement effect of EOC was better than that of DLI, which was consistent with the results of behavioral and biochemical analysis. Therefore, these results suggested that EOC and DLI could improve learning and memory impairments in D-gal-induced aging mice in part due to its protective effect on the hippocampus, and EOC was superior to DLI in improving learning and memory impairment.

More importantly, EOC and DLI affect the balance of oxidation and antioxidant systems, thus improving learning and memory impairment. MDA is the key index of lipid peroxidation caused by oxidative damage, which indirectly increases ROS. SOD and GSH-Px are considered as the first line of defense of antioxidant system, directly inhibiting ROS and free radical (Shi et al., 2014). After EOC and DLI treatment, MDA in hippocampus of mice decreased significantly, SOD and GSH-PX increased significantly, indicating that EOC and DLI could significantly restore redox balance, and the improvement effect of EOC is better than that of DLI. Therefore, inhibiting oxidative stress caused by ROS may be a potential way to improve learning and memory impairments.

In previous studies, EOC and DLI have not been found to improve learning and memory impairment in D-gal-induced mice. Normally, the interaction between Keap1 and Nrf2 makes Nrf2 in a relatively inhibited state. However, in the presence of a large number of reactive oxygen species, Nrf2 dissociates from Keap1 and regulates the transcription and translation activities of antioxidant factors (such as HO-1) and ensure redox homeostasis (Kobayashi and Yamamoto, 2005). The present results show that after EOC-H and DLI-H treatment, the expression of Nrf2 and HO-1 increased, especially EOC-H, and the expression of Keap1 decreased significantly. EOC and DLI can partially protect the brain from oxidative damage through the antioxidant signal pathway of Nrf2 to improve learning and memory impairment. NF-κB is another protein activated by oxidative stress and involved in regulating various cell activities, including inflammatory response (Garcia et al., 2000; Yang et al., 2015). Under normal conditions, IκκB and NF-κB binds to each other in the cytoplasm, preventing NF-κB enters the nucleus and destroys DNA (Chen et al., 2019). However, in response to several pathogenic factors, NF-κB is dissociated and activated and enters the nucleus, initiating transcription of downstream factors and promoting inflammatory responses by regulating pro-inflammatory cytokines IL-1β and TNF-α (Jeong et al., 2017; Li et al., 2020; Saber et al., 2020). Not only IL-1 β and TNF- α are important pro-inflammatory factors, but also studies have shown high levels of IL-1β and TNF-α which can accelerate the aging process. The western blot results showed that EOC-H and DLI-H could down-regulate the protein expression of Iκκβ, NF-κB, IL-1β and TNF-α. Notably, EOC and DLI significantly reduced the production of TNF-α and IL-1β, especially EOC-H. Therefore, this study suggests that EOC and DLI play a neuroprotective role through antioxidant stress, which may be related to NF-κB pathway.

## 5 Conclusion

In conclusion, this study confirmed that EOC and DLI could improve the learning and memory impairment of accelerated brain aging mice induced by D-galactose, enhance their antioxidant capacity, increase the protein expression of Nrf2 and HO-1, and inhibit Iκκβ、NF-κB、IL-1β and TNF-α, and the neuroprotective effect of EOC was better than that of DLI. Our findings suggest that EOC will help alleviate age-related learning and memory disorders.

## Data Availability

The original contributions presented in the study are included in the article/supplementary material, further inquiries can be directed to the corresponding authors.
